# Venous blood gas kinetics and acid–base correction during incremental intermittent hemodialysis in dogs with advanced renal failure

**DOI:** 10.14202/vetworld.2026.667-677

**Published:** 2026-02-23

**Authors:** Sachin Sachin, Randhir Singh, Raj Sukhbir Singh, Gurpreet Singh Preet

**Affiliations:** 1 Department of Veterinary Medicine, Guru Angad Dev Veterinary and Animal Sciences University, Ludhiana-141 004, Punjab, India; 2 Department of Teaching Veterinary Clinical Complex, Guru Angad Dev Veterinary and Animal Sciences University, Ludhiana-141 004, Punjab, India

**Keywords:** acid–base balance, canine renal failure, hemodialysis, incremental intermittent hemodialysis, metabolic acidosis, renal replacement therapy, venous blood gas, veterinary nephrology

## Abstract

**Background and Aim:**

Acid–base disturbances, particularly metabolic acidosis, are common in dogs with advanced renal failure and contribute substantially to morbidity and prognosis. Intermittent hemodialysis (IHD) is increasingly used when conventional therapy fails; however, physiological monitoring parameters for incremental intermittent hemodialysis (i-IHD) in dogs remain poorly defined. Venous blood gas (VBG) analysis offers a safer and more practical alternative to arterial sampling, yet its utility during i-IHD has not been systematically evaluated. This study aimed to characterize longitudinal changes in VBG and hemato-biochemical parameters before and after consecutive i-IHD sessions in dogs with renal failure.

**Materials and Methods:**

In this prospective observational study, 45 client-owned dogs with severe azotemia (serum creatinine >5 mg/dL) due to acute kidney injury (AKI) stage IV–V or chronic kidney disease (CKD) stage IV were enrolled. All dogs underwent three consecutive i-IHD sessions with stepwise increases in treatment intensity. Venous blood samples were collected immediately before and after each session for VBG analysis, including pH, bicarbonate (HCO_3_^-^), total carbon dioxide (TCO_2_), partial pressures of carbon dioxide (pCO_2_) and oxygen (pO_2_), base excess, anion gap, and cerebral oxygen saturation, along with hemato-biochemical profiling. Dialysis adequacy was assessed using Kt/V, urea reduction ratio, and creatinine reduction ratio. Pre- and post-dialysis values were compared using paired statistical analyses.

**Results:**

Dogs exhibited mild-to-moderate metabolic acidosis before i-IHD. Across all sessions, i-IHD produced a consistent and significant correction of acid–base imbalance, evidenced by normalization of pH and marked increases in HCO_3_^-^ and TCO_2_ (p < 0.01). A modest but significant rise in pCO_2_ accompanied bicarbonate repletion, while the anion gap remained within the lower borderline range, indicating non-anion gap metabolic acidosis. Significant reductions in blood urea nitrogen and creatinine (approximately 25%–40% per session) confirmed effective solute clearance, with adequacy indices improving progressively across sessions. Electrolyte abnormalities, particularly hyperkalemia, were effectively corrected.

**Conclusion:**

I-IHD effectively restores acid–base and biochemical homeostasis in dogs with advanced renal failure. Serial VBG monitoring provides clinically meaningful, session-wise information and represents a practical tool for guiding i-IHD without the risks of arterial sampling.

## INTRODUCTION

The kidneys play a central role in maintaining metabolic homeostasis in dogs and receive approximately 20% of the cardiac output. In canine patients, the most severe forms of renal injury include acute kidney injury (AKI) and chronic kidney disease (CKD) [1]. AKI is characterized by a rapid decline in renal function, resulting in the accumulation of uremic waste products, disturbances in fluid balance, and electrolyte and acid–base abnormalities [2]. In contrast, CKD is a progressive disorder marked by a gradual and irreversible loss of renal functional capacity. This progressive decline disrupts cellular metabolism and ultimately compromises cardiovascular function [3]. CKD is further associated with a reduced glomerular filtration rate (GFR), persistent albuminuria, or both [4].

Intermittent hemodialysis (IHD) represents an advanced adjunctive therapeutic option when conservative management fails in cases of severe azotemia, intoxications, fluid overload, fluid imbalance, and AKI [5]. IHD is also recommended for the removal of selected toxins, even in the presence of kidney injury. In such circumstances, a comprehensive evaluation is required to determine the necessity of dialysis, taking into account factors such as toxin concentration, toxin half-life, and the availability of specific antidotes, as well as the anticipated efficacy of hemodialysis in toxin removal [6]. Despite its clinical benefits, hemodialysis is costly and may be financially prohibitive for many pet owners, particularly given the uncertainty of prognosis. Therefore, reliable prognostic tools are essential for predicting outcomes in dogs with AKI or CKD when considering hemodialysis [7]. The systemic accumulation of uremic toxins in renal failure can precipitate multi-organ dysfunction, thereby increasing mortality risk in affected patients [8].

Under normal physiological conditions, the kidneys are fundamental to the regulation of acid–base balance through tubular reabsorption and the generation of new bicarbonate (HCO_3_^-^). Serum HCO_3_^-^ is routinely included in biochemical panels for the assessment of renal disease and is commonly used as a surrogate marker of systemic acid–base status. Metabolic acidosis (MA) is frequently observed in both human and veterinary patients with CKD, with a higher prevalence in advanced stages of the disease [9]. A serum HCO_3_^-^ concentration below 22 mmol/L is generally considered indicative of MA, which typically develops when GFR declines below 30 mL/min. In patients with CKD, MA is associated with poorer renal outcomes and increased mortality. Chronic MA may arise from pathophysiological alterations involving the circulatory system, nutritional status, inflammation, and bone mineral metabolism [10]. Although HCO_3_^-^ concentration is widely used for the diagnosis and management of MA in CKD, there is no universal consensus regarding its optimal clinical application. Base excess (BE) serves as an additional important indicator of metabolic acid–base disturbances, reflecting the magnitude of excess acid or base in plasma [11]. Alongside HCO_3_^-^ BE has been shown to be a valuable predictor of mortality in critically ill patients and those with acute kidney injury [12].

Despite the increasing clinical use of IHD in dogs with AKI and CKD, there remains a critical lack of evidence regarding the physiological responses to an incremental treatment strategy. Most veterinary studies have focused primarily on solute clearance, survival outcomes, or single-session biochemical changes, with limited attention to dynamic acid–base adaptation across consecutive dialysis sessions. In particular, validated monitoring parameters for incremental intermittent hemodialysis (i-IHD) are poorly defined, and the role of venous blood gas (VBG) analysis as a longitudinal, session-wise monitoring tool has not been systematically investigated in dogs. Arterial blood gas analysis, although informative, is invasive and often impractical in routine clinical settings, whereas VBG offers a safer and more feasible alternative. However, robust data describing how key VBG variables, such as pH, HCO_3_^-^ BE, and pCO_2_, evolve during stepwise i-IHD are lacking. Furthermore, the predominance and behavior of non-anion gap MA during i-IHD, as well as its relationship with electrolyte shifts and dialysis adequacy indices, remain insufficiently characterized in canine patients. This knowledge gap limits the development of evidence-based protocols for optimizing dialysis intensity and ensuring physiological stability during i-IHD.

The present study aimed to evaluate longitudinal changes in VBG and hemato-biochemical parameters in dogs with AKI and CKD undergoing i-IHD. Specifically, the study sought to assess pre- and post-dialysis alterations in pH, HCO_3_^-^, BE, pCO_2_, pO_2_, TCO_2_, and anion gap across three consecutive i-IHD sessions, and to relate these changes to electrolyte balance and dialysis adequacy indices. By characterizing session-wise acid–base kinetics using VBG analysis, this study aimed to determine the clinical utility of VBG as a practical monitoring tool for guiding individualized i-IHD protocols and improving the safety and physiological outcomes of dogs with advanced renal failure.

## MATERIALS AND METHODS

### Ethical approval and informed consent

The study was conducted in accordance with the guidelines of the Institutional Animal Ethics Committee (IAEC) of Guru Angad Dev Veterinary and Animal Sciences University, Ludhiana, Punjab, India, and was approved during the 71st IAEC meeting (Approval No. GADVASU/2024/IAEC/71/21, dated January 09, 2024).

All dogs included in the study were client-owned animals presented for clinical management of advanced renal failure, and i-IHD was performed solely on therapeutic grounds. Prior to enrollment, informed written consent was obtained from all owners after detailed counseling regarding the nature of the disease, indications for i-IHD, procedural steps, potential risks and complications, anticipated benefits, financial implications, and the need for repeated sampling and monitoring.

Given the life-saving and interventional nature of hemodialysis, withdrawal of consent after vascular catheter placement was not permitted to ensure patient safety and continuity of care. No experimental interventions beyond standard clinical practice were performed, and all procedures were carried out with due consideration for animal welfare.

This study was conducted over a period of 1 year and included 45 client-owned dogs. i-IHD sessions were performed on days 01, 02, and 05 of the treatment protocol.

### Study duration and location

This prospective observational clinical study was performed over a period of 1 year from January 2024 to January 2025 at the Dialysis Unit, Multi-speciality Veterinary Hospital, Guru Angad Dev Veterinary and Animal Sciences University, Ludhiana, Punjab, India.

### Study population and characteristics of patients

The study was conducted using a consecutive case enrollment strategy over 45 client-owned dogs irrespective of age, breed, body weight, and sex (Table 1). The selection criteria of dogs were based on the following: serum creatinine level >5 mg/dL with uremic signs, oliguria/anuria, electrolyte imbalances, or non-response to conventional medicinal therapy and diagnosis of AKI stage IV or V and CKD stage IV based on modified IRIS guidelines [13].

**Table 1:** Characterization of patients undergoing incremental intermittent hemodialysis.

Parameter	Number
Age (Mean ± SEM)	7.36 ± 2.04
Range	2–12 years
Body weight (Mean ± SEM)	30.91 ± 8.32
Range	12–52 kg
Sex	
Male	36 (80)
Female	9 (20)
Proportion	
AKI	15
CKD	30
Breed distribution	
American Bully	6
American Bull Terrier	2
Beagle	1
Bull Terrier	1
Chow Chow	1
Gaddi	2
Golden Retriever	8
German Shepherd	8
Labrador Retriever	11
Non-descript	3
Rottweiler	1
Saint Bernard	1

AKI = Acute kidney injury, CKD = Chronic kidney disease, SEM = Standard error of the mean.

Dogs were classified as having AKI stage IV–V or CKD stage IV based on clinical history, illness duration, laboratory findings (including urine concentrating ability), and imaging studies. The study included 15 dogs with AKI [AKI stage IV non-oliguric (9 dogs), AKI stage IV oliguric (3 dogs), and AKI stage V oliguric (3 dogs)] and 30 dogs with CKD (IRIS CKD IV). Dogs with severe coagulopathy, uncontrolled sepsis, terminal illness, severe cardiac disease, or inability to tolerate or have a dialysis catheter placed were excluded from the study.

### VBG analysis

Whole venous blood was manually collected in 2 mL heparinized syringes. The syringe’s barrel was coated with sodium heparin, and the excess heparin was forcefully expelled. All visible air bubbles were immediately expelled, and the syringes were sealed to prevent atmospheric gas exchange. Venous blood samples were collected from a cephalic or saphenous peripheral vein.

The sample was processed immediately before and after i-IHD for three consecutive sessions. Blood samples were analyzed immediately (within 120 s) using a bedside point-of-care blood gas analyzer (EPOC® Blood Analysis System, Siemens Healthineers, Erlangen, Germany). The analyzer was calibrated according to standard quality assurance protocols, and all variables were measured and recorded at 37°C. As per the manufacturer’s protocol, the analyzer performed automatic electronic calibration and sensor verification with each single-use test card prior to sample analysis.

Measured parameters included pH, partial pressure of oxygen (pO_2_, mmHg), partial pressure of carbon dioxide (pCO_2_, mmHg), bicarbonate (HCO_3_^-^, mmol/L), BE (mmol/L), cerebral oxygen saturation (cSO_2_), total carbon dioxide (TCO_2_), anion gap (AGap), and potassium-corrected anion gap (AGapk).

Calculated parameters included sodium (Na), potassium (K), chloride (Cl), ionized calcium, hematocrit (Hct), hemoglobin (Hb), glucose, lactate, blood urea nitrogen, uric acid, and creatinine (Cr).

### i-IHD protocol and adequacy of dialysis

The Fresenius 4008S (ng) dialysis machine (Fresenius Medical Care, Bad Homburg, Germany) was used for dialysis of the selected canine patients. This machine controls extracorporeal circulation, regulates the supply, flow, and temperature of the dialysate solution, and mixes the dialysate concentrate with heated aerated water to the desired proportion (1 part dialysate concentrate to 34 parts water). The dialysate solution was continuously monitored and maintained at the appropriate temperature and conductivity.

The machine was equipped with a blood pump adjustable to different speeds to alter blood flow through the dialyzer. The pump automatically disconnected and ceased blood flow if any malfunction was detected by the monitoring system. For anticoagulation, heparin was infused into the extracorporeal circuit at a rate of 50 IU/kg/h. An ultrafiltration facility enabled precise fluid removal calculation, prediction, and control during dialysis. A built-in audio-visual alarm system promptly notified the veterinary dialysis specialist in the event of any system malfunction.

Blood tubing (AV-SETFMC PAED-R, Fresenius Medical Care, Bad Homburg, Germany) with an inner diameter of 6.4 mm and a filling volume of 117 mL was used to establish extracorporeal circulation. The tubing consisted of arterial and venous limbs distinguished by red and blue ports, respectively. Blood passed through the dialyzer and was collected in a bubble trap with a clot filter before returning to the patient. Infusions could be administered into either the arterial or venous line.

The dialysate used was a commercially available two-component system. Part A (Hemobicarb®, Hindustan Lifecare Limited, Thiruvananthapuram, India) was a concentrated acidic solution containing dextrose and electrolytes, and Part B was a sodium bicarbonate solution (Bibag®, Fresenius Medical Care, Bad Homburg, Germany). The final dialysate composition at a 1:34 dilution contained sodium 135.00 mmol/L, potassium 2.00 mmol/L, calcium 1.75 mmol/L, magnesium 0.50 mmol/L, chloride 107.25 mmol/L, glucose 5.05 mmol/L, and acetate 3.50 mmol/L.

Dialyzer size was selected based on body weight to minimize extracorporeal volume. The circuit was primed with sterile saline followed by heparinized saline (5000 IU/5 mL at 50 IU/kg), with excess discarded before venous connection. The low-flux dialyzer FX8 (Fresenius Medical Care, Bad Homburg, Germany) utilized a helicon membrane with a surface area of 1.4 m².

**Figure 1:**
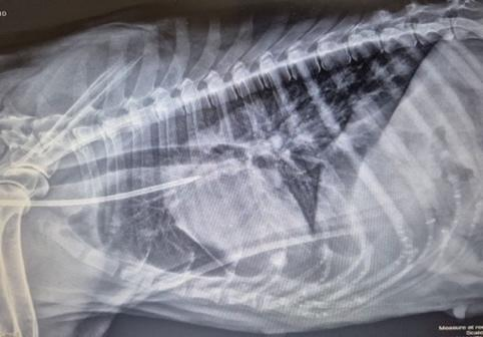
Lateral thoracic radiograph showing correct positioning of the double-lumen hemodialysis catheter, with the catheter tip located at the junction of the cranial vena cava and right atrium, ensuring uninterrupted extracorporeal blood flow during dialysis.

A temporary double-lumen dialysis catheter (11.5 Fr, 19 cm; Stone Medical Devices Private Limited, Haryana, India) was placed in the right jugular vein under local anesthesia using 10% lignocaine spray (Lox®, Neon Laboratories Ltd., Mumbai, India). Catheter position at the cranial vena cava–right atrium junction was confirmed radiographically (Figure 1) [14].

**Figure 2:**
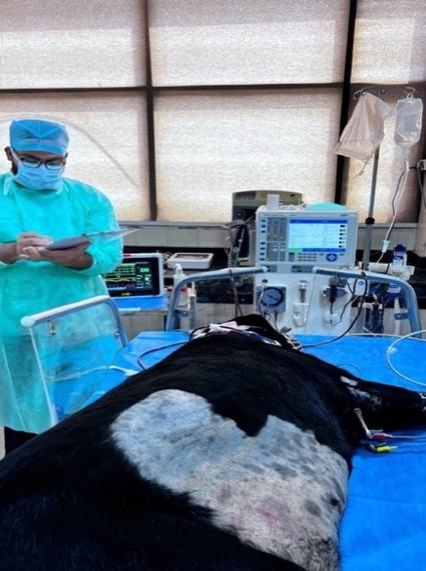
Continuous clinical and technical monitoring of a dog during an ongoing incremental intermittent hemodialysis session, including patient positioning, vascular access management, and dialysis equipment surveillance.

Vital parameters were continuously monitored, and blood pressure was assessed every 15 min using the Doppler method (Figure 2). Hypotension was managed by ultrafiltration adjustment, intravenous fluid boluses, and pharmacologic support. Isotonic saline was infused using an automatic infusion pump (Infusia VP7, Fresenius Kabi, Bad Homburg, Germany). Dialysis was performed incrementally over three sessions with blood flow increased from 2 to 7 mL/kg/min and duration extended from 2 to 4 h, while dialysate flow was maintained at 500 mL/min.

Dialysis adequacy was assessed using Kt/V, URR, and CrRR according to standard formulas and prescribed treatment intensity [15].

### Post-dialysis care and supportive therapy

After the third i-IHD session, the catheter was removed under aseptic conditions. The insertion site was cleansed with povidone–iodine, and sterile gauze soaked with tranexamic acid (Zakshot®, Carus Laboratories Pvt. Ltd., Mumbai, India) was applied with firm pressure for 10–15 min.

Supportive therapy included pantoprazole (Prasopheg®, Aquitas Healthcare Pvt. Ltd., Mumbai, India), maropitant (Pet-omitic®, Veko Care Pvt. Ltd., Hyderabad, India), ampicillin (Roscillin®, Sun Pharmaceutical Industries Ltd., Mumbai, India), and enrofloxacin (Floxidin®, Intervet India Pvt. Ltd., Pune, India). Post-dialysis supplementation included L-carnitine (One-Carni®, Sinder K, Mumbai, India) and an essential amino acid infusion (Nephrosteril® 7%, Fresenius Kabi, Bad Homburg, Germany). All dogs were maintained on a renal canine diet (Farmina Pet Foods, Russo Mangimi S. p.A., Nola, Italy).

### Statistical analysis

Data were analyzed using SAS statistical software version 9.2 (SAS Institute Inc., Cary, NC, USA). Paired *t*-tests were used to examine differences between pre- and post-treatment blood gas and hemato-biochemical parameters for each session. The Shapiro–Wilk test confirmed approximate normal distribution of paired differences, justifying parametric analysis. Statistical significance was set at p < 0.05.

## RESULTS

### Demographic characteristics of dogs undergoing i-IHD

Among dogs undergoing i-IHD, males predominated (36/45; 80%), whereas females accounted for 20%. The mean body weight of dogs in the i-IHD cohort was 30.91 ± 8.32 kg. Most cases originated from urban areas (71.11%; 32/45), while 28.88% were from rural regions. The majority of dogs were older than 8 years (20/45; 44.4%), followed by those aged 6.1–8 years (16/45; 35.6%). Younger dogs were less frequently represented, with 17.8% aged 3.1–6 years and only 2.2% aged 1–3 years.

### Changes in VBG parameters during i-IHD

The VBG parameters underwent substantial alterations during i-IHD, as summarized in Table 2. Across all three i-IHD sessions, a significant correction of acid–base equilibrium was observed, resulting in marked changes in blood gas variables. The most prominent alterations included changes in pH, depletion of HCO_3_^-^, and reduction in pCO_2_. The primary abnormality identified was MA with partial respiratory compensation.

**Table 2:** Comparison of blood gas changes in dogs undergoing incremental intermittent hemodialysis (i-IHD).

Parameter (Reference interval)	IHD Session I (2 h)			IHD Session II (3 h)			IHD Session III (4 h)		
		
	Pre-IHD	Post-IHD	p-value	Pre-IHD	Post-IHD	p-value	Pre-IHD	Post-IHD	p-value
pH (7.31–7.42)	7.29 ± 0.01	7.44 ± 0.00	p = 0.000	7.37 ± 0.01	7.46 ± 0.01	p = 0.000	7.40 ± 0.00	7.49 ± 0.00	p = 0.000
pCO_2_ (29–42 mmHg)	32.71 ± 0.88	35.92 ± 0.91	p = 0.000	33.16 ± 0.66	37.16 ± 0.85	p = 0.000	33.61 ± 0.81	36.14 ± 0.97	p = 0.000
pO_2_ (85–95 mmHg)	43.20 ± 1.86	33.44 ± 1.23	p = 0.000	37.11 ± 1.69	29.73 ± 1.28	p = 0.000	38.89 ± 1.43	34.11 ± 1.85	p = 0.000
HCO₃⁻ (17–24 mmol/L)	16.18 ± 0.74	24.28 ± 0.59	p = 0.000	20.44 ± 0.62	27.71 ± 0.42	p = 0.000	21.38 ± 0.51	27.30 ± 0.45	p = 0.000
BE (ecf) (−3 to +3 mmol/L)	−9.26 ± 1.06	0.09 ± 0.64	p = 0.000	−3.86 ± 0.84	4.53 ± 0.42	p = 0.000	−2.07 ± 0.91	4.22 ± 0.53	p = 0.000
cSO_2_ (40–90 %)	73.06 ± 2.03	64.43 ± 2.30	p = 0.000	68.22 ± 2.31	61.02 ± 2.00	p = 0.000	70.62 ± 2.61	65.06 ± 2.31	p = 0.04
TCO_2_ (13–27 mmol/L)	17.36 ± 0.94	23.52 ± 0.65	p = 0.000	20.36 ± 0.67	27.20 ± 0.63	p = 0.000	21.51 ± 0.69	26.11 ± 0.64	p = 0.000
AGap (12–24 mmol/L)	10.42 ± 0.61	9.55 ± 0.51	p = 0.18	9.57 ± 0.51	9.71 ± 0.50	p = 0.77	9.08 ± 0.51	9.66 ± 0.54	p = 0.18
AGapk (12–25 mmol/L)	13.60 ± 0.58	12.57 ± 0.49	p = 0.12	12.88 ± 0.44	12.91 ± 0.46	p = 0.96	12.37 ± 0.45	12.57 ± 0.54	p = 0.65

AGap = Anion gap, AGapk = Anion gap of potassium, BE = Base excess, cSO_2_ = Cerebral oxygen saturation, ecf = Extracellular fluid, HCO₃⁻ = Bicarbonate, pCO_2_ = Partial pressure of carbon dioxide, pH = Potential of hydrogen ion, pO_2_ = Partial pressure of oxygen, RI = Reference interval, TCO_2_ = Total carbon dioxide.

**Figure 3:**
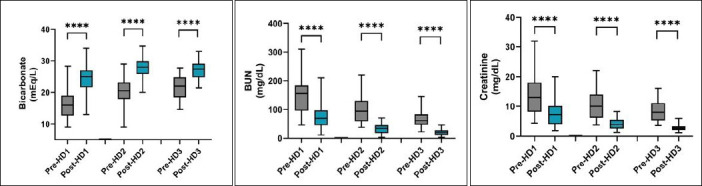
Box-and-whisker plots illustrating changes in blood urea nitrogen (BUN), serum creatinine, and bicarbonate concentrations before and after successive incremental intermittent hemodialysis sessions (HD1–HD3) in dogs. A significant decrease in BUN and creatinine concentrations and a significant increase in bicarbonate concentration were observed following each i-IHD session. Data are presented as median, interquartile range, and minimum–maximum values.**** indicates highly significant differences between pre- and post-dialysis values.

The mean pH transitioned from mild-to-moderate acidemia during the pre-dialysis period and progressively approached normal values during the post-dialysis period. Session I demonstrated normalization of pH, indicating effective correction of systemic acidosis. In subsequent sessions, pH values shifted toward the upper physiological limit, accompanied by a moderate increase in HCO_3_^-^ concentration. Post-dialysis pH increased from 7.44 in Session I to 7.49 in Session III, while HCO_3_^-^ increased from 16.18 to 27.30 mmol/L, highlighting the effective buffering action of bicarbonate-based dialysate (Figure 3).

Although the increase in pH from 7.44 to 7.49 appears numerically small, it reflects a clinically relevant shift toward alkalinization, indicating efficient neutralization of excess hydrogen ions by extracellular buffering systems, predominantly bicarbonate. This response is consistent with the expected physiological outcome of hemodialysis, which facilitates removal of uremic toxins and restoration of acid–base equilibrium through bicarbonate-rich dialysate.

The anion gap, typically ranging from 12 to 24, remained at a lower borderline range, suggesting the presence of non-anion gap MA. This pattern is indicative of HCO_3_^-^ loss, renal tubular acidosis, or hyperchloremic or uremic acidosis. During all three i-IHD sessions, pCO_2_, increased significantly, which may be attributed to HCO_3_^-^ repletion and compensatory mechanisms enhancing blood buffering capacity.

Moderate hypoxemia was observed during the post-dialysis period, potentially related to intradialytic fluid shifts and interactions between blood and the artificial dialyzer membrane. Although remaining within the normal range, cSO_2_, showed a declining trend across the three i-IHD sessions. This trend may be associated with alterations in hemoglobin–O_2_, affinity during extracorporeal circulation and fluid dynamic changes during i-IHD. Following dialysis, TCO_2_, increased significantly (p < 0.01), further supporting improvement in acid–base balance. Similarly, pCO_2_, exhibited a modest but significant increase (p < 0.01), likely reflecting improved ventilation–perfusion equilibrium.

### Hemato-biochemical alterations associated with i-IHD

In this cohort of 45 dogs, i-IHD produced consistent and clinically meaningful changes in biochemical and hematological parameters (Table 3). Post-dialysis potassium concentrations decreased markedly (p < 0.001), whereas calcium concentrations increased significantly (p < 0.001). Chloride concentrations showed a consistent decline after each session (p < 0.001). Hematocrit and hemoglobin values were significantly reduced following dialysis (p < 0.05), suggesting mild hemodilution. Glucose concentrations decreased significantly (p < 0.01), while lactate concentrations remained unchanged. No significant differences were observed in sodium concentrations between pre- and post-dialysis measurements.

**Table 3:** Comparison of hemato-biochemical changes in dogs undergoing incremental intermittent hemodialysis (i-IHD).

Parameter (Reference interval)	IHD Session I		Paired t-test	IHD Session II		Paired t-test	IHD Session III		Paired t-test
		
	Pre-IHD	Post-IHD		Pre-IHD	Post-IHD		Pre-IHD	Post-IHD	
Na (142–152)	140.80 ± 0.92	139.71 ± 0.54	t = 1.51, p = 0.13	138.27 ± 0.79	139.36 ± 0.53	t =−1.55, p = 0.12	138.16 ± 0.89	137.00 ± 2.23^a^	t = 0.51, p = 0.61
K (3.9–5.1)	4.07 ± 0.17	2.94 ± 0.08	t = 9.46, p = 0.00	3.75 ± 0.11	3.01 ± 0.07	t = 8.22, p = 0.00	3.87 ± 0.12	3.10 ± 0.07	t =10.44, p = 0.00
iCa	1.03 ± 0.03	1.20 ± 0.03	t =−4.77, p = 0.00	1.13 ± 0.02^a^	1.35 ± 0.02	t =−8.56, p = 0.00	1.14 ± 0.02^b^	1.37 ± 0.02^b^	t =−9.35, p = 0.00
Cl (110–124)	112.36 ± 1.16	105.00 ± 0.57	t = 6.17, p = 0.00	108.47 ± 0.89	103.40 ± 0.99	t = 5.25, p = 0.00	107.00 ± 0.95^b^	103.16 ± 0.57	t = 5.65, p = 0.00
HCT	31.71 ± 1.35	29.81 ± 1.26	t = 3.27, p = 0.00	29.79 ± 1.40	28.62 ± 1.32	t = 2.58, p = 0.01	28.78 ± 1.33	26.56 ± 1.19	t = 5.07, p = 0.00
Hb (12–16)	10.65 ± 0.44	10.17 ± 0.44	t = 2.56, p = 0.01	10.09 ± 0.43	9.72 ± 0.42	t = 2.90, p = 0.00	9.72 ± 0.43	9.01 ± 0.40	t = 5.87, p = 0.00
Glucose (76–119)	115.93 ± 5.27	100.98 ± 2.87	t = 2.98, p = 0.00	108.51 ± 2.52	100.80 ± 2.56	t = 2.86, p = 0.00	106.64 ± 2.49	97.47 ± 3.35	t = 3.25, p = 0.00
Lactate (<2.5)	1.38 ± 0.23	1.58 ± 0.25	t =−0.92, p = 0.36	1.32 ± 0.14	1.15 ± 0.16	t = 1.18, p = 0.24	1.48 ± 0.18	1.90 ± 0.26	t =−1.75, p = 0.08
BUN (8–28)	147.67 ± 9.07	73.07 ± 5.87	t =16.57, p = 0.00	97.13 ± 6.39	33.53 ± 2.51	t =14.23, p = 0.00	66.64 ± 4.09	21.02 ± 1.65	t =15.80, p = 0.00
Urea	53.42 ± 3.17	26.55 ± 1.96	t =14.09, p = 0.00	34.19 ± 2.37	13.59 ± 1.26	t =12.33, p = 0.00	24.73 ± 1.96	8.33 ± 0.84	t =11.70, p = 0.00
Cr (0.5–1.7)	13.98 ± 0.95	7.31 ± 0.58	t = 14.45, p = 0.00	10.20 ± 0.66	4.10 ± 0.28	t = 13.12, p = 0.00	8.52 ± 0.47	2.75 ± 0.16	t = 15.16, p = 0.00

Means with different superscripts (a, b) within columns differ significantly (p < 0.05). BUN = Blood urea nitrogen, Cl = Chloride, Cr = Creatinine, Hb = Hemoglobin, HCT = Hematocrit, iCa = Ionized calcium, K = Potassium, Na = Sodium

### Trends in hematocrit and hemoglobin over treatment sessions

A gradual decline in both hematocrit and hemoglobin values was observed over the course of treatment. Hematocrit decreased consistently from 31.71 ± 1.35% to 29.81 ± 1.26% (t = 3.27, p = 0.00), then to 28.62 ± 1.32% (t = 2.58, p = 0.01), and finally to 26.56 ± 1.19% (t = 5.07, p = 0.00). Similarly, hemoglobin concentration declined from 10.65 ± 0.44 g/dL to 10.17 ± 0.44 g/dL (t = 2.56, p = 0.01), then to 9.72 ± 0.42 g/dL (t = 2.90, p = 0.00), and subsequently to 9.01 ± 0.40 g/dL (t = 5.87, p = 0.00). Overall, both parameters demonstrated a steady and statistically significant reduction, indicating a progressive decline in red blood cell indices during the observation period.

### Dialysis prescription, adequacy, and complications

Dialysis was performed in accordance with Cowgill’s established protocols [15]. Treatment intensity for Sessions I, II, and III was prescribed based on pre-dialysis BUN concentrations. To minimize the risk of complications such as dialysis disequilibrium syndrome and hypotension, URR and CrRR targets were set at 40%–50%, 50%–65%, and >65% for Sessions I, II, and III, respectively. Correspondingly, Kt/V targets were defined as 0.60–0.80, 0.81–1.20, and >1.20 for Sessions I, II, and III.

Blood pump speed was maintained at 2 mL/kg/min during Session I, increased to 5 mL/kg/min in Session-II, and further increased to 7 mL/kg/min in Session III. Major complications observed during the study included shivering (n = 08), vomiting (n = 04), hypotension (n = 01), and ventricular premature complexes (n = 01). All complications were effectively managed using established medical management protocols.

### Overall impact of i-IHD on physiological parameters

This study clearly demonstrated that i-IHD significantly improved VBG parameters, electrolyte balance, and uremic solute clearance in dogs with renal dysfunction. A progressive and statistically significant correction of MA was evident across the three dialysis sessions, as reflected by increasing pre- and post-dialysis pH, HCO_3_^-^, and BE values.

## DISCUSSION

### Acid–base alterations during i-IHD

This study demonstrated significant variation in acid–base parameters among dogs undergoing i-IHD, with the most prominent changes observed in pH, pCO_2_, and HCO_3_^-^ levels. The use of VBG analysis as a longitudinal monitoring tool during i-IHD represents a practical and clinically relevant advancement in veterinary nephrology. Overall, i-IHD resulted in a consistent and progressive correction of MA and improvement in acid–base parameters without significant alteration of the anion gap, underscoring its efficacy and safety in managing uremic acid–base disturbances in dogs.

Comparable findings were reported by de Azevedo *et al*. [16], who observed increased blood pH, sodium bicarbonate, and BE in dogs undergoing IHD in bypass mode. Kraut and Madias [17] previously reported that non-anion gap acidosis is more prevalent in patients with renal impairment. The predominance of NAGMA observed in the present study likely reflects impaired renal acid excretion and reduced HCO_3_^-^ reclamation rather than accumulation of unmeasured organic acids. In dogs with advanced renal dysfunction, tubular impairment and decreased ammonia genesis contribute to hyperchloremic MA. The relatively low and stable anion gap throughout the study supports this mechanism. Incremental hemodialysis facilitates gradual correction of this acid–base disorder by restoring HCO_3_^-^ balance without inducing major shifts in unmeasured anions. This condition is associated with increased protein catabolism, muscle atrophy, skeletal dysfunction, renal osteodystrophy, and cardiovascular complications.

### Comparison with human and veterinary studies

A similar study in human hemodialysis patients by Rindaha *et al*. [18] demonstrated the most pronounced effects on pO_2_ and SO_2_, variables. Likewise, Marano *et al*. [19] reported that MA was the most common acid–base disturbance in CKD patients undergoing dialysis, affecting 22 (41.5%) individuals, followed by respiratory alkalosis in five (9.4%) and respiratory acidosis in two (3.8%) patients. Acid–base disequilibrium is a central feature of renal failure in both dogs and humans. Yamamoto *et al*. [20] identified blood pH as a critical prognostic indicator of mortality in patients receiving hemodialysis. Similarly, Wieliczko and Małyszko [21] reported marked fluctuations in pH, HCO_3_^-^, lactate, and pCO_2_ before and after hemodialysis sessions.

These observations are consistent with previous veterinary studies demonstrating the effectiveness of hemodialysis in correcting acid–base disturbances [22, 23]. In the present study, BE normalized from markedly negative values to mildly positive levels, indicating steady resolution of acidemia. TCO_2_ closely paralleled HCO_3_^-^ trends, while both the anion gap and potassium-corrected anion gap remained largely unchanged, further supporting a diagnosis of non-anion gap MA. The mild post-dialysis increase in pCO_2_ may reflect attenuation of compensatory hyperventilation, whereas the decline in pO_2_ was clinically negligible and likely attributable to transient intradialytic ventilation–perfusion shifts.

### Cerebral oxygenation and physiological adaptation

A mild declining trend in cSO_2_ was observed across i-IHD sessions, which may reflect transient hemodynamic or perfusion-related adaptations during extracorporeal circulation. Importantly, no clinical signs of neurological dysfunction were observed during or after dialysis. Therefore, venous cSO_2_ values should be interpreted cautiously, particularly in the absence of overt neurological deficits. Further studies incorporating continuous perfusion monitoring are warranted to clarify the clinical significance of these findings.

### Interpretation of acid–base analysis frameworks

Physicochemical models of acid–base balance, particularly the Stewart framework, describe bicarbonate as a dependent variable influenced by strong-ion difference, total weak acids, and pCO_2_ [24]. The strong-ion gap has subsequently been proposed as a refined indicator of unmeasured ions compared with the traditional anion gap [25]. Despite these conceptual advances, HCO_3_^-^ concentration, BE, and the anion gap remain the most widely used and clinically intuitive acid–base indices in veterinary practice, especially in dogs undergoing hemodialysis. In this context, bicarbonate-based parameters directly reflect therapeutic correction of MA and are routinely used to guide dialysis prescription and monitoring. Moreover, accurate calculation of the strong-ion gap requires comprehensive strong-ion profiling and albumin correction, which were beyond the scope of this study. Consequently, a bicarbonate-centered analytical approach was retained while acknowledging the complementary insights provided by physicochemical models [26].

### Electrolyte and hematological responses to i-IHD

Electrolyte profiling revealed efficient potassium removal, consistent with observations by Cowgill and Francey [27], along with consistent correction of hypocalcemia, likely attributable to calcium-containing dialysate. The large and reproducible reductions in serum potassium across all three sessions reflect effective intradialytic K^+^ removal and align with established dialysis physiology, emphasizing the need for careful potassium monitoring to avoid rapid fluctuations that may compromise cardiac stability.

Electrolyte changes beyond potassium were predictable and clinically relevant. Post-dialysis increases in calcium likely resulted from dialysate composition and redistribution effects, whereas consistent reductions in chloride may reflect correction of uremic hyperchloremia or dilutional changes associated with ultrafiltration. Sodium concentrations remained stable. Hematocrit and hemoglobin declined modestly but significantly following dialysis, consistent with intradialytic hemodilution and fluid shifts. Similar findings were reported by Polzin and Ross [28]. Anemia in dogs undergoing IHD may result from blood loss, heparin anticoagulation, mechanical hemolysis, and extracorporeal circuit interactions [29].

Blood contact with synthetic materials within catheters, bloodlines, and dialyzers may promote platelet activation and consumption [30], a process that can be exacerbated by heparin use. Heparin-induced thrombocytopenia is a recognized dialysis complication [31], highlighting the importance of platelet monitoring and consideration of alternative anticoagulation strategies.

### Metabolic effects, complications, and clinical outcomes

Despite effective clearance of nitrogenous waste and correction of fluid, acid–base, and electrolyte imbalances, i-IHD may induce a negative protein balance. Glucose concentrations showed modest reductions after dialysis, whereas lactate levels remained stable, indicating that i-IHD does not predispose to lactic acidosis. Reduced glucose levels likely reflect removal of circulating glucose and insulin by the extracorporeal circuit, supporting the need for peri-dialytic glucose monitoring, particularly in dogs with altered glycemic control. Stable lactate concentrations suggest preserved systemic perfusion and absence of significant anaerobic metabolism during i-IHD.

Adverse events were infrequent and generally manageable, with shivering and vomiting being the most common complications. These findings are consistent with extracorporeal blood cooling, solute shifts, and uremia-associated gastrointestinal responses. Episodes of hypotension and ventricular premature complexes were rare and resolved with standard medical management, indicating good procedural safety.

### Uremic solute clearance and clinical relevance

Failure of renal toxin elimination leads to uremia in dogs with CKD [32]. In this study, BUN, urea, and creatinine concentrations declined significantly across all sessions, confirming effective nitrogenous waste removal and dialysis adequacy [15, 33]. The consistency of these trends supports the clinical utility of an incremental dialysis strategy, allowing gradual correction and physiological adaptation. Unlike earlier veterinary studies focusing primarily on solute clearance or survival, the present study provides a dynamic assessment of acid–base adaptation over time.

Although a formal responder–non-responder analysis was not performed, most dogs showed consistent improvement in acid–base and biochemical parameters with limited inter-individual variability. Collectively, these findings indicate that i-IHD is a safe, adaptable, and effective approach for managing acid–base, electrolyte, and metabolic disturbances in dogs with renal disease. Importantly, this study highlights the value of a longitudinal within-patient design using repeated VBG measurements, validating VBG as a practical monitoring tool during dialysis and reducing reliance on arterial sampling in canine patients.

## CONCLUSION

This study demonstrated that i-IHD produced a consistent and progressive correction of MA in dogs with advanced renal dysfunction, as evidenced by significant improvements in VBG parameters across three consecutive sessions. Post-dialysis increases in pH, HCO_3_^-^, BE, and TCO_2_ along with a modest rise in pCO_2_, indicated effective buffering and partial normalization of acid–base homeostasis without inducing major shifts in the anion gap, supporting the predominance of non-anion gap MA. Concurrently, i-IHD resulted in marked reductions in BUN, urea, and creatinine, confirming effective uremic solute clearance and improving dialysis adequacy indices. Electrolyte disturbances, particularly hyperkalemia and hypocalcemia, were consistently corrected, while hematological changes reflected mild, expected hemodilution. Adverse events were infrequent, manageable, and did not compromise procedural safety.

The findings highlight the clinical utility of VBG as a reliable, minimally invasive, and practical monitoring tool during i-IHD. Routine session-wise VBG assessment can assist clinicians in tracking acid–base kinetics, tailoring dialysis intensity, and minimizing complications associated with rapid correction. The incremental approach allows physiological adaptation, reduces the risk of DDS and hemodynamic instability, and offers a pragmatic strategy for managing dogs with AKI and CKD in clinical settings.

Key strengths include the prospective design, longitudinal within-patient evaluation across three consecutive i-IHD sessions, and comprehensive integration of VBG, hemato-biochemical parameters, and dialysis adequacy indices. The study provides one of the first detailed characterizations of acid–base adaptation during i-IHD in dogs, thereby addressing an important gap in veterinary nephrology.

The study was limited by its single-center design and the inclusion of a mixed AKI–CKD population, which may influence generalizability. The absence of arterial blood gas comparison, long-term follow-up and survival analysis also restricts broader interpretation of physiological and outcome-based effects.

Future multicenter studies with larger cohorts, stratified AKI- and CKD-specific analyses, and longer follow-up periods are warranted. Comparative evaluation of VBG versus arterial blood gas analysis, incorporation of outcome-based endpoints, and assessment of alternative anticoagulation strategies would further refine i-IHD protocols. Exploration of nutritional and protein balance effects during prolonged i-IHD is also recommended.

Overall, i-IHD is a safe, adaptable, and effective modality for correcting acid–base, electrolyte, and metabolic disturbances in dogs with renal disease. Serial VBG monitoring offers a clinically meaningful approach to guide individualized dialysis management while reducing procedural risks. These findings support the integration of i-IHD and VBG-guided monitoring into routine veterinary nephrology practice.

## DATA AVAILABILITY

All the generated data are included in the manuscript. The supplementary data can be made available from the corresponding author upon request.

## AUTHORS’ CONTRIBUTIONS

Singh R. was responsible for the clinical management and initiation of hemodialysis in the study cohort. Preet G. S. and Sachin performed blood gas assessments and assisted in designing and prescribing the hemodialysis protocol. Sachin led data collection, analysis, manuscript drafting, and follow-up evaluations. Singh R. S. provided overall supervision of the study, contributed to study design, data interpretation, and critically revised the manuscript. All authors have read and approved the final version of the manuscript.
